# In Memoriam Werner Waldhäusl, 27 September 1937–22 May 2025

**DOI:** 10.1007/s00125-025-06489-1

**Published:** 2025-07-09

**Authors:** Michael Roden

**Affiliations:** 1 Department of Endocrinology and Diabetology, Medical Faculty and University Hospital Düsseldorf, Heinrich Heine University, Düsseldorf, Germany; 2 Institute for Clinical Diabetology, German Diabetes Center, Leibniz Center for Diabetes Research at Heinrich Heine University Düsseldorf, Düsseldorf, Germany; 3 German Center for Diabetes Research (DZD), Partner Düsseldorf, München-Neuherberg, Germany


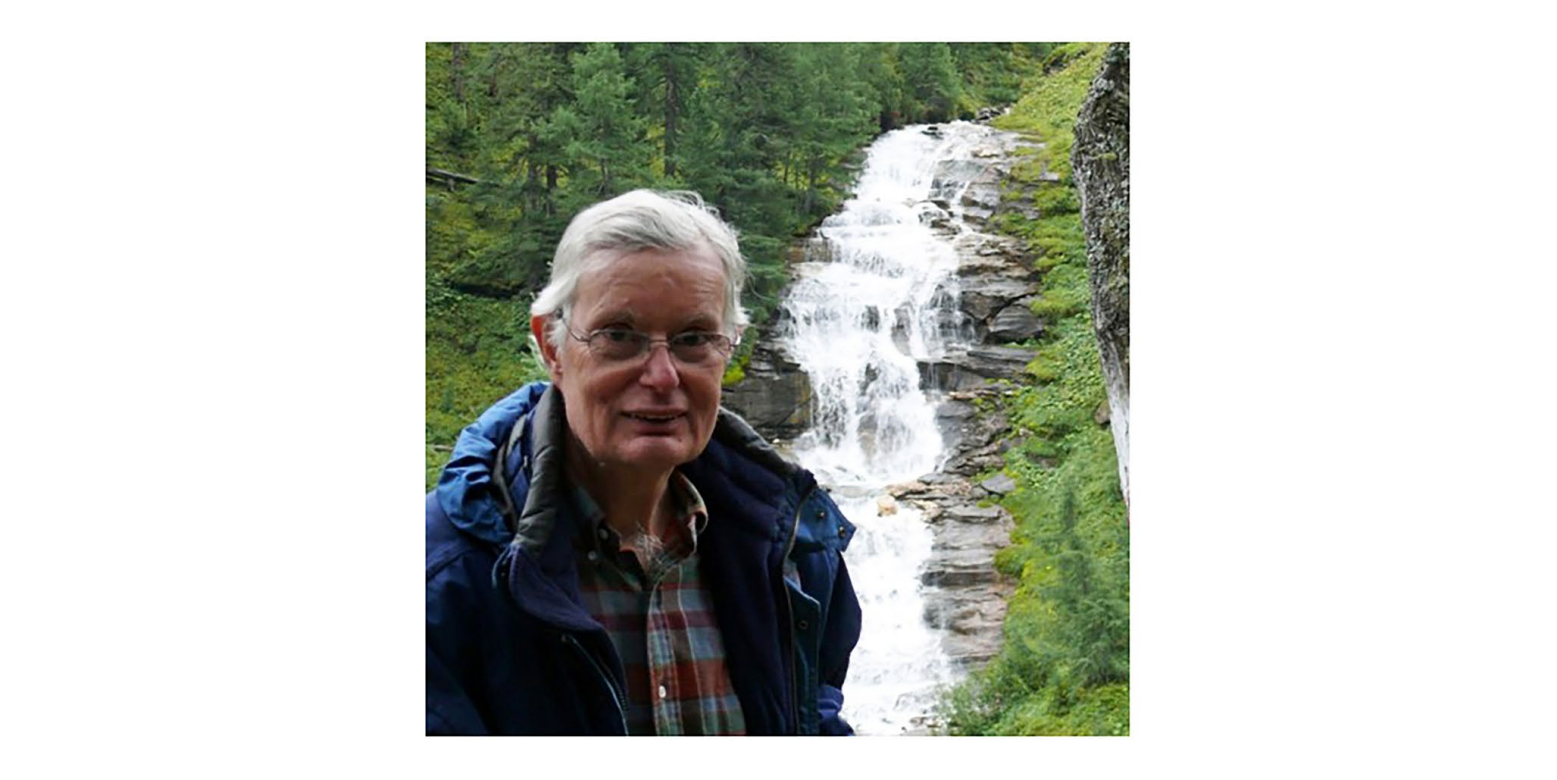


A leading figure in European diabetology, o. Univ. Prof. em. Dr. Werner Waldhäusl passed away in May in Vienna, Austria, leaving behind his wife, Marianne; his sons, Martin and Christoph; his brother, Peter; and their families.

Werner graduated from high school with highest honours, received a doctorate in medicine in Vienna, and started his internship in a local hospital outside Vienna before switching gears to experimental pathology—and ultimately to internal medicine—at the 1st Medical University Clinics and General Hospital of Vienna in 1965. Upon returning from a research fellowship in hypertension research with Professor Jerome W. Conn at the University of Michigan Medical School, MI, USA, he founded the Division of Endocrinology and Diabetology at the 1st Medical University Clinics in Vienna, which he chaired from 1987. In 1992, he became chair and director of the Department of Medicine III and its Division of Endocrinology and Metabolism at the newly established university hospital. He received numerous awards, including the Claude Bernard Prize from the EASD, the Paul Langerhans Medal from the German Diabetes Association (DDG) and the Von Mering Gold Medal from the German Diabetes Center (DDZ). He also served as president and honorary member of several scientific associations, including Vice President of EASD (1983–1984) and Editor-in-Chief of *Diabetologia* (1998–2003).

Werner’s research began with investigations into endocrine blood pressure regulation, during which he detected a novel pressor principle from pig serum, findings that were published in *Nature* [1], before contributing to the isolation of renin with Professor Conn, whose signed photo he kept in his office throughout his academic career. He soon also became interested in the human physiology of the interplay between insulin secretion and glucose disposal, using the invasive hepatic arterial and venous catheterisation technique. With this approach, he studied splanchnic substrate metabolism, finding that only half of the glucose ingested during a standard 75 g oral glucose load is temporarily retained by the splanchnic bed, and also measured splanchnic C-peptide production in humans [2]. Werner also showed that post-prandial hyperglycaemia in people with obesity and type 2 diabetes is mainly due to peripheral, rather than hepatic, insulin resistance [3], which could be later proven in detail using non-invasive methods. These studies began to shed light on differences in tissue-specific insulin resistance. His studies on the effect of initial hypo-osmolar fluids during severe hyperglycaemia have important clinical implications [4], emphasising the need for rehydration rather than high-dose insulin during diabetic ketoacidosis—an established standard today, but hotly debated 25 years ago.

This was before I met Werner, who, upon taking over leadership of the 1st Medical University Clinics in Vienna, completely changed the recruitment system for young fellows—from years-long, not entirely transparent waiting lists to an interview-based system conducted by him and his colleagues. To cut a long story short, two colleagues and I were allowed to join his group and to undergo demanding training for the next decade. Werner always served as a role model of a physician-scientist, exemplifying perfect manners, highest self-discipline and tireless interest in focused research, which is best illustrated by citing him in his role as Editor-in-Chief: “*Diabetologia* will be pleased to publish such reports, short and clear even if negative, as long as the questions asked are new and the methodology used sound” [5]. But most importantly, he was always interested in new concepts, even if he was not convinced at the beginning, which I remember when my group started to develop magnetic resonance spectroscopy to quantify hepatic energy turnover [6].

Throughout his career, he was a dedicated follower of a healthy lifestyle, best reflected by his legendarily frugal lunches—one small sliced apple. He repeatedly emphasised the need for motivation, including financial incentives, to combat obesity and diabetes through lifestyle modification, which he continued to examine under real-life conditions when he became head of a rehabilitation clinic in Austria’s countryside following his time as Vice Rector of the University of Veterinary Medicine Vienna. After his retirement, he remained active, continued to advise his previous coworkers and contributed his comments in international meetings, until about 2 years ago when a sudden accident stopped his public and private activities—an immediate caesura also for his beloved wife and family.

Werner Waldhäusl shall be remembered as one of the great teachers and physician-scientists in internal medicine and diabetology.
